# Supplementary material for: Splice-Junction-Based Mapping of Alternative Isoforms in the Human Proteome
Source: Cell Rep. Author manuscript; Available in PMC 2020 Jan 15. (PMC6961840; doi:10.1016/j.celrep.2019.11.026)
Supplement: 2 [file NIHMS1546469-supplement-2.zip › DF1/PXD006675/TricuspidValve/TricuspidValve_3_TNS1_PHNPADILLHPTGEEDEGK.pdf]

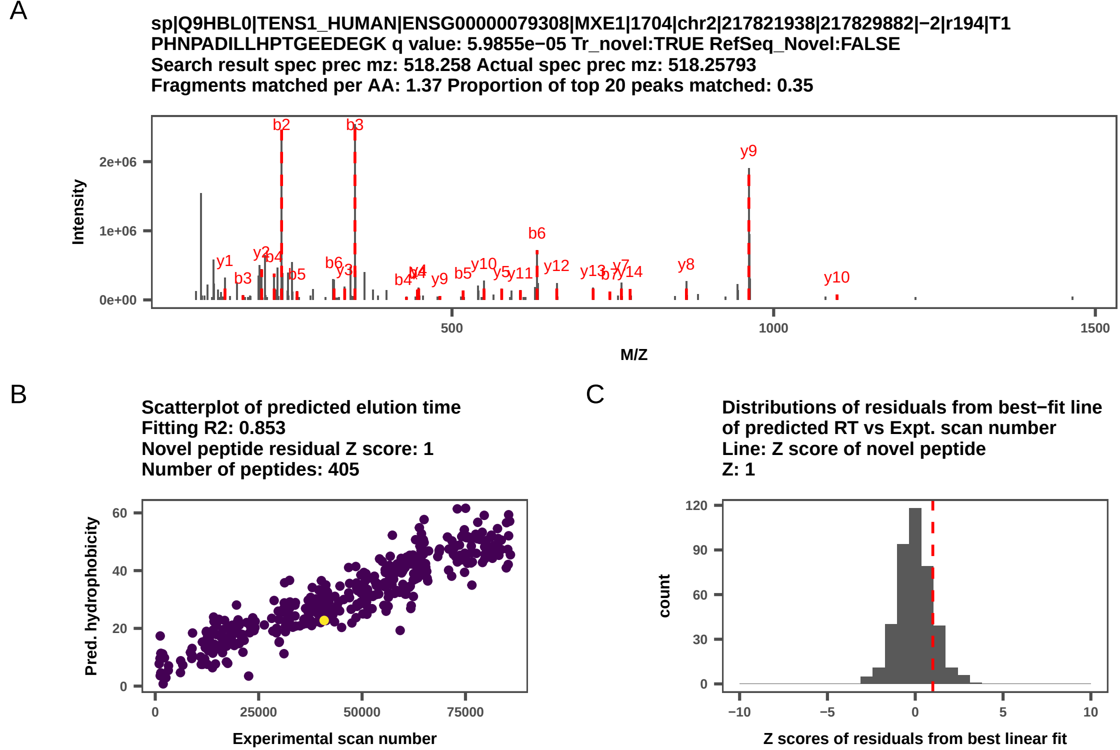
A
sp|Q9HBL0|TENS1_HUMAN|ENSG00000079308|MXE1|1704|chr2|217821938|217829882|−2|r194|T1
PHNPADILLHPTGEEDEGK q value: 5.9855e−05 Tr_novel:TRUE RefSeq_Novel:FALSE
Search result spec prec mz: 518.258 Actual spec prec mz: 518.25793
Fragments matched per AA: 1.37 Proportion of top 20 peaks matched: 0.35
Intensity
2e+06
1e+06
0e+00
b2
b3
y9
b6
y1
b3
y2
b4
b5
b6
y3
b4
y4
y9
b5
y10
y5
y11
y12
y13
b7
y7
y14
y8
y10
500
1000
1500
M/Z
B
Scatterplot of predicted elution time
Fitting R2: 0.853
Novel peptide residual Z score: 1
Number of peptides: 405
Pred. hydrophobicity
60
40
20
0
0
25000
50000
75000
Experimental scan number
C
Distributions of residuals from best−fit line
of predicted RT vs Expt. scan number
Line: Z score of novel peptide
Z: 1
count
120
90
60
30
0
−10
−5
0
5
10
Z scores of residuals from best linear fit
